# Stabilization of β-Galactosidase on Modified Gold Nanoparticles: A Preliminary Biochemical Study to Obtain Lactose-Free Dairy Products for Lactose-Intolerant Individuals

**DOI:** 10.3390/molecules26051226

**Published:** 2021-02-25

**Authors:** Asim Muhammed Alshanberi, Rukhsana Satar, Shakeel Ahmed Ansari

**Affiliations:** 1 Department of Community Medicine and Pilgrims Health Care, Umm Alqura University, Makkah 24382, Saudi Arabia; amshanberi@uqu.edu.sa; 2 Department of Biochemistry, School of Medicine, Batterjee Medical College for Sciences and Technology, Jeddah 21442, Saudi Arabia; 3 Division of Biochemistry, Ibn Sina National College for Medical Studies, Jeddah 22421, Saudi Arabia; rukhsanasatar@ibnsina.edu.sa

**Keywords:** β-galactosidase, surface modification, gold nanoparticles, stability

## Abstract

The unique chemical, optical, and electrical characteristics of nanoparticles make their utilization highly successful in every field of biological sciences as compared to their bulk counterpart. These properties arise as a result of their miniature size, which provides them an excellent surface area-to-volume ratio, inner structure, and shape, and hence increases their surface characteristics. Therefore, this study was undertaken to engineer gold nanoparticles (AuNPs) for improving their catalytic activity and stability in biotechnological processes. The characterization of AuNPs was performed by XRD, UV spectra, and TEM. The synthesized AuNPs were surface-modified by polyvinyl alcohol (PVA) for binding the enzyme in excellent yield. The developed immobilized enzyme system (PVA-AuNPs-β-galactosidase) displayed pH optima at pH 7.0 and temperature optima at 40 °C. Moreover, the stability of PVA-AuNPs-β-galactosidase was significantly enhanced at wider pH and temperature ranges and at higher galactose concentrations, in contrast to the free enzyme. β-galactosidase bound to PVA-modified AuNPs exhibited greater operational activity, even after its sixth reuse. The developed nanosystem may prove useful in producing lactose-free dairy products for lactose-intolerant patients.

## 1. Introduction

Enzyme immobilization is necessary for biosensor development and for constructing reactors for industrial applications [1]. This technology is required for stabilizing the enzymes against chemical and physical denaturants, for resistance to product-mediated inhibition, and for offering repeated use in industrial processes [2]. Several techniques that have been employed previously for immobilization of β-galactosidases include entrapment [3], crosslinking [4], adsorption, and bioaffinity-based procedures [5,6,7,8]. Covalent immobilization of enzymes on matrices is perhaps a desirable approach to achieve stronger immobilization, unlike physical adsorption, without affecting bulk properties. Several researchers have previously discussed the immobilization of enzymes on various biomaterials using this approach [9,10,11].

Covalent binding of enzyme immobilization is attracting a lot of attention due to the related stability enhancement [12]. Moreover, this procedure provides oriented immobilization to enzymes that facilitate good expression of activity and reusability [13,14]. This method of enzyme immobilization retains very high enzyme activity, as no modification/distortion occurs at the active site of enzyme. Since the active site is less hindered by the nanomatrix, the steric accessibility allows greater access to incoming substrates and outgoing products. Moreover, affinity binding offers very mild, controlled adsorption of biocatalysts onto the supports and is likely to be of continuing value for the immobilization of sensitive biocatalysts [15,16].

Recently, nanoparticles have also attracted the attention of researchers to improve traditional enzyme immobilization. Nanoparticle-based enzyme immobilization is an enigma that deserves special attention, as it provides a greater surface area for binding higher amounts of enzyme to the matrix, and prevents unfolding of protein and permits greater flexibility for the conformational changes required for enzyme activity [17,18]. The other advantages involved with its use include continuous operations, catalyst recycling, enhanced stability, easy separation from the reaction mixture, possible modulation of the catalytic properties, and much easier prevention of microbial growth [19,20].

The advancement in the synthesis procedure makes it possible to formulate nanoparticles with precise control over physico-chemical and optical properties that are desired for specific clinical or biological applications [21]. Surface modification technology has further added impetus to the specific applications of nanoparticles by imparting them the desirable characteristics [22]. Such strategies involve introduction of ligands on the surface of nanoparticles via simple chemical reactions to stabilize the nanoparticles against agglomeration and providing compatibility with other phases in a self-organized manner [23]. In this regard, gold nanoparticles (AuNPs) have enabled scientists to develop biofuel cells, enzyme-based biosensors, and industrial applications due to their biocompatibility and stability within harsh environments [24]. Furthermore, the introduction of various bio/chemical ligands to their surface encourages their additional applications in a variety of biomedical and biotechnological applications [25].

β-galactosidase (Enzyme Commission 3.2.1.23) is one such industrially important enzyme that possesses both hydrolytic and transgalactosylation activity, which is required for obtaining lactose-free products and producing lacto-sucrose, respectively. Hence, this enzyme is of paramount importance in dairy industry for obtaining lactose-free milk and galacto-oligosaccharides [26,27].

Hence, this study was undertaken to observe the stability of β-galactosidase immobilized on modified AuNPs by accessing its activity against chemical and physical denaturants. For this purpose, AuNPs were synthesized and characterized, and their surface was modified with polyvinyl alcohol (PVA) for binding β-galactosidase. Reusability studies of immobilized β-galactosidase were also evaluated.

## 2. Results and Discussion

### 2.1. Selection of AuNPs for the Current Study

AuNPs have been employed previously for constructing drug delivery vehicles, biosensors, solar cells, and lubricants, and as antimicrobial agents [28,29,30]. However, they are less utilized for immobilizing enzymes, which are of therapeutic and industrial importance. The shape and size and interaction with various bio/chemical ligands offer AuNPs unique physico-chemical properties, which make them highly favorable in biomedical applications over other nanoparticles. Moreover, owing to the broad specificity for the substrate, poly-functionality and availability of β-galactosidase from different sources allow their utilization in various biotechnological processes [31]. Hence, the current research was undertaken to synthesize and modify the surface of AuNPs by PVA for observing their efficacy in β-galactosidase stabilization.

### 2.2. Synthesis and Characterization of AuNPs

**Figure 1:**
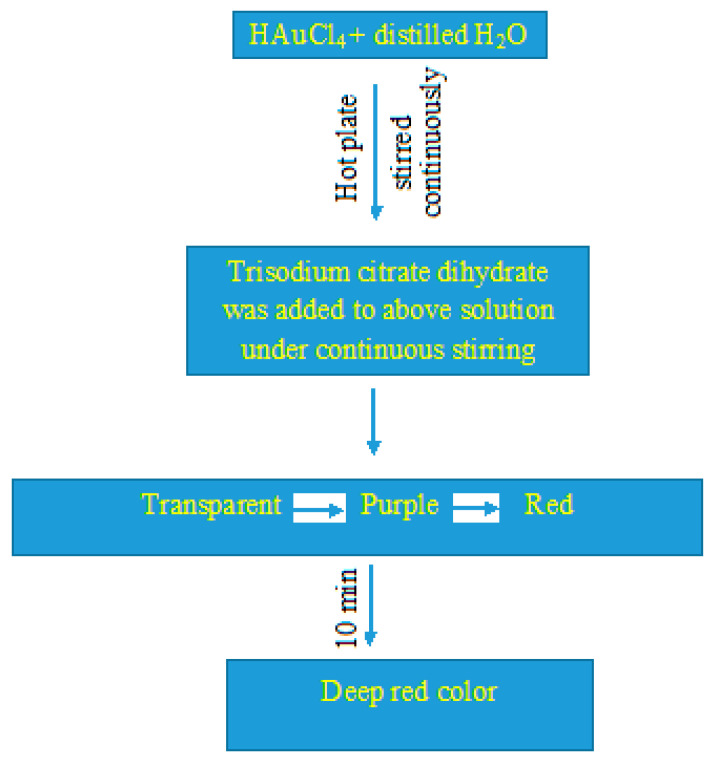
Schematic representation for synthesizing the gold nanoparticles (AuNPs).

Figure 1 represents a flowchart for synthesizing AuNPs, which is indicated by the development of deep red color.

**Figure 2:**
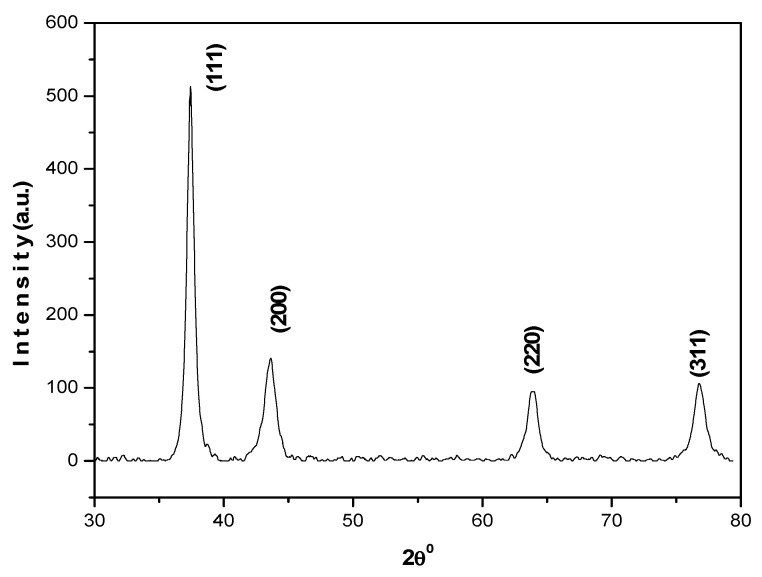
XRD pattern of AuNPs.

The method used to formulate AuNPs herein was easy and favored their synthesis without aggregation, in high yield and with low preparation cost. XRD analysis revealed peaks (2θ) at 38.18°, 44.39°, 64.57° and 77.54° (Figure 2).

**Figure 3:**
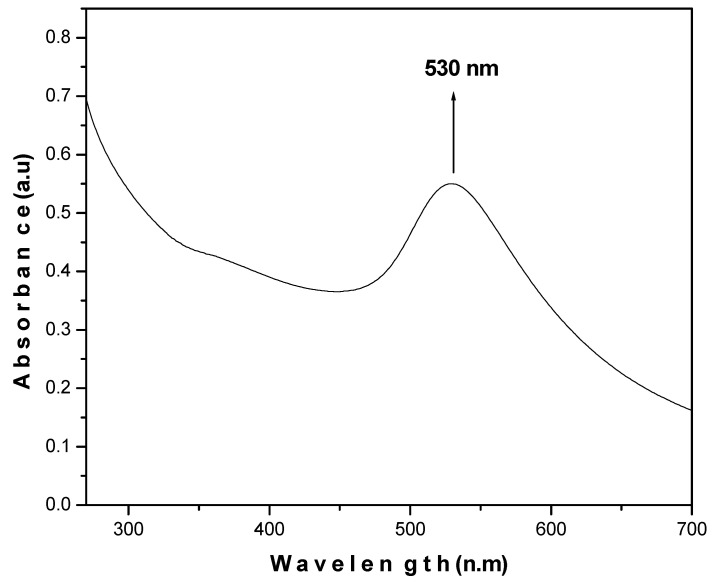
UV-Vis spectrum of AuNPs.

The results obtained are in agreement with the JCPDS value (040784) and support the formation of a polycrystalline face-centered cubic structure of AuNPs. UV-visible absorption spectra demonstrate the optical and structural characteristics of metallic nanoparticles due to the absorption bands associated with their precise diameter and aspect ratio. It was revealed that AuNPs absorb light at around 530 nm, which exhibits the characteristic absorption peak of AuNPs (Figure 3).

**Figure 4:**
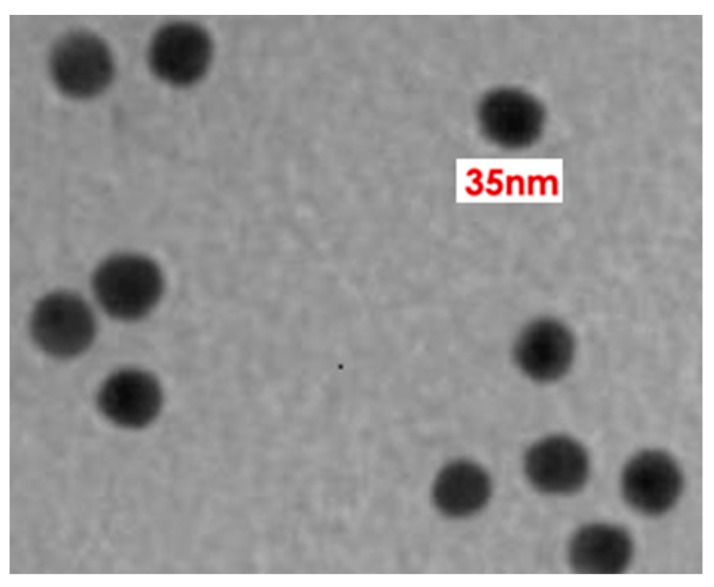
TEM analysis of AuNPs.

TEM images, obtained by a dwell time of 2 s, which were averaged over eight frames, revealed the size of synthesized AuNPs as 35–40 nm (Figure 4). These results are in good agreement with previous reports [32,33].

### 2.3. Surface Functionalization of AuNPs via PVA for Stabilizing β-Galactosidase

Modifying the surface of nanoparticles is a widely employed technique for imparting specific properties to them in industrial applications. PVA was used in the current study to modify the surface of AuNPs because of its excellent durability and mechanical stability, low biodegradability, negligible toxicity, and lack of side effects for enzyme reactions [34].

### 2.4. Stabilization of PVA-AuNPs-β-Galactosidase Against Physical and Chemical Denaturation

**Figure 5:**
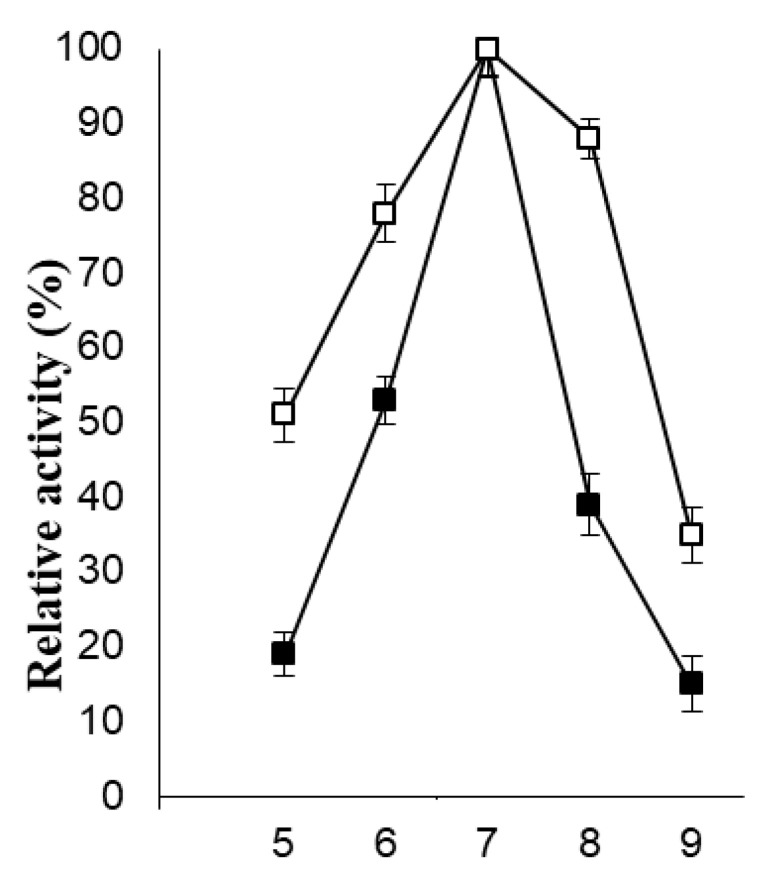
pH activity profiles for soluble and immobilized β-galactosidase. The symbols show soluble (■) and immobilized (□) β-galactosidase.

The industrial utility of immobilized β-galactosidase is favored over soluble enzymes due to their excellent recyclable stability, volume-specific biocatalyst loading, and simplified downstream processing. Satar and co-workers previously modified graphene for the immobilization of β-galactosidase for improved galacto-oligosaccharide (GOS) synthesis. This approach exhibited an increase in Km of the immobilized enzyme without a noticeable change in the V_max_ value. Maximum GOS content was also achieved by the immobilized enzyme at higher temperatures as compared to the soluble β-galactosidase [35]. Nonetheless, the immobilization procedure may result in conformational changes, which might affect the catalytic efficiency of enzymes. It should be noted that in order to design an exceptionally effective immobilized enzyme nanobiocatalyst for biotechnological applications, its stability should be excellent at lower and higher temperature ranges, and in both acidic and basic pH ranges [36]. The pH activity profiles for soluble β-galactosidase and the enzyme bound to PVA-modified AuNPs are shown in Figure 5.

**Figure 6:**
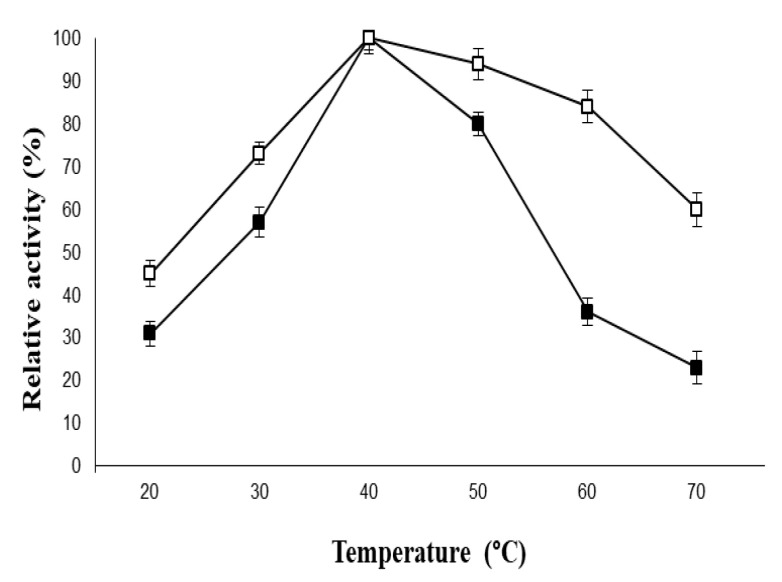
Temperature activity profiles of soluble and immobilized β-galactosidase. For symbols, please refer to the legend of Figure 5.

The developed immobilized β-galactosidase system exhibited a pH optimum similar to the free enzyme i.e., pH 7.0. However, a significant broadening in the pH activity profiles was observed for the immobilized β-galactosidase as compared to its native counterpart. Moreover, the free enzyme showed 53% activity at pH 6.0, whereas the immobilized β-galactosidase retained 78% activity under similar experimental conditions. This could be attributed to the fact that greater alteration/distortion was produced in the tertiary structure of soluble β-galactosidase in highly acidic and basic solutions as compared to the β-galactosidase immobilized on PVA-modified AuNPs. Similarly, the loss in enzyme activity was greater for the free enzyme at higher temperatures as compared to the enzyme bound to the modified AuNPs (Figure 6) due to denaturation of enzyme molecules, which ruptured the polypeptide chain. Hence, it can be concluded that there was no substantial change in the secondary interactions in the conformational equilibrium of the enzyme and PVA-modified AuNPs during immobilization.

**Figure 7:**
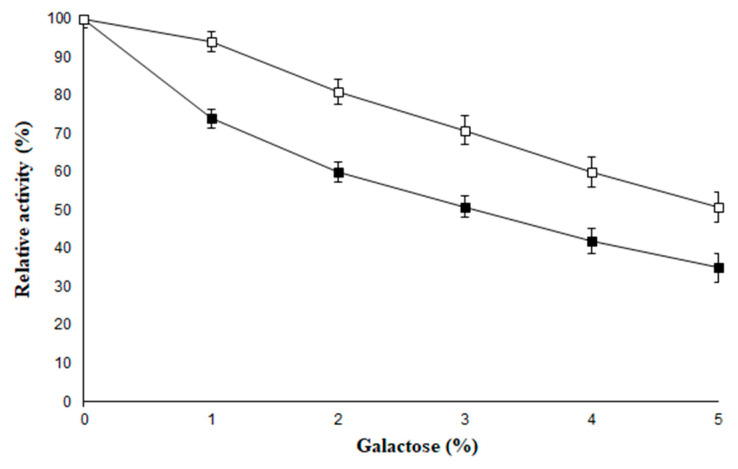
Effect of galactose on soluble and immobilized β-galactosidase. For symbols, please refer to the legend of Figure 5.

Galactose is one of the end products of the reaction that involves the hydrolysis of lactose, and acts as a competitive inhibitor for β-galactosidase in varied concentration ranges [37]. This study demonstrates the effectiveness of PVA-AuNPs-β-galactosidase against higher concentrations of galactose (Figure 7). The soluble β-galactosidase showed 51% activity in the presence of 3.0% galactose, while the enzyme attached to PVA-AuNPs-β-galactosidase exhibited much higher enzyme activity (i.e., 71%) at the same concentration of galactose. The results for the immobilized enzyme exhibited promising resistance to the inhibition mediated by galactose as compared to its soluble counterpart.

### 2.5. Operational Stability of PVA-AuNPs-β-Galactosidase

**Figure 8:**
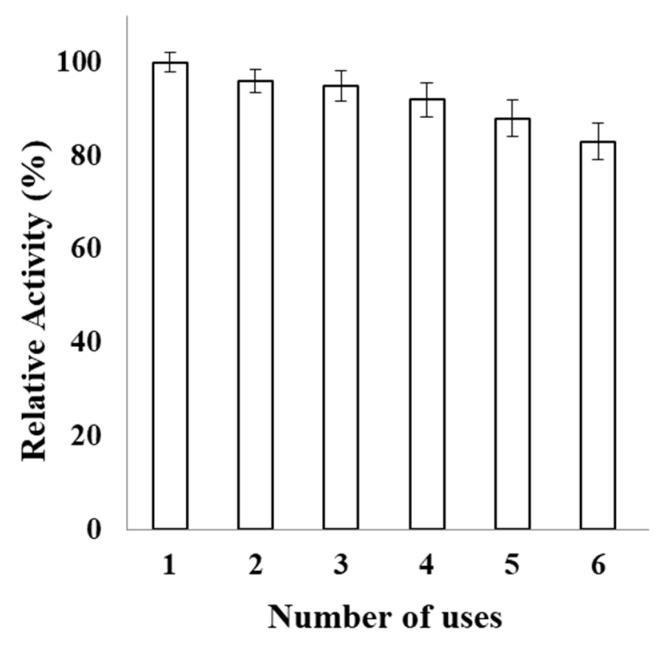
Reusability of immobilized β-galactosidase. Reusability of immobilized β-galactosidase (100 µL) was monitored for 6 successive days. The aliquots were taken in triplicates and were assayed for the remaining percent activity. The activity determined on the first day was taken as control (100%) for the calculation of remaining activity after each use.

Enzyme engineering through immobilization on surface-modified nanomatrices via multi-subunit covalent binding, entrapment, and adsorption is a preferred approach for improving enzyme properties, such as inhibition by reaction products, activity, specificity, and stability [38]. The consequent reuse of β-galactosidase bound to PVA-modified AuNPs is shown in Figure 8. IβG retained 83% activity even after its sixth repeated use; hence, it can provide economic benefits during its industrial application.

## 3. Materials and Methods

*Kluyveromyces lactis* β-galactosidase and ONPG were obtained from Sigma Aldrich, USA. All of the reagents were prepared in deionized water and the sterility of glassware was achieved by incubating for at 37 °C for one day.

### 3.1. Synthesis and Characterization of AuNPs

AuNPs were synthesized from HAuCl_4_ in solution form by modifying the procedure slightly as described earlier [39]. Briefly, 0.1 mM HAuCl_4_ was mixed with distilled water (20 mL) and kept on a hot plate (80 °C) under stirring conditions. Trisodium citrate dihydrate (5%) was added to the solution under stirring conditions. It transformed from transparent to a purple color initially and finally to red. This change in color was followed by a deep red color after 10 min, which indicated the formation of AuNPs. The XRD pattern for the synthesized AuNPs was obtained using an X-ray diffractometer (Rigaku-Miniflex) with CuK_α_ (λ = 1.54056 Å) radiations as the X-ray source in the 2θ range from 20^o^ to 80^o^ with scan rate of 2^o^ per min at room temperature. A UV-Vis spectrophotometer UV-1800 and an Sl-210 double beam UV visible spectrophotometer were used for obtaining the UV-Vis spectra. Morphological characterization of the synthesized AuNPs was recorded by TEM (JEOL 2010F) at 200 keV.

### 3.2. Surface Modification of AuNPs by PVA

The deionized water was used to wash the AuNPs synthesized above and retrieved by centrifuging for 15 min at 1000 rpm. The obtained nanoparticles were suspended in 2.0% PVA for 4 h in a shaker at 200 rpm. The PVA-modified AuNPs were washed thrice with deionized water and centrifuged for removing the traces of PVA [40]. The resulting modified AuNPs were subsequently washed with potassium phosphate buffer (100 mM, pH 7.0) for binding the enzyme.

### 3.3. β-Galactosidase Binding on PVA-Modified AuNPs

β-galactosidase (2000 units) was incubated overnight with PVA-functionalized AuNPs under slow stirring at 32 °C. The unbound/loosely bound enzyme was eliminated by washing it thrice with potassium phosphate buffer (100 mM, pH 7.0). The PVA-AuNPs-β-galactosidase obtained thus was suspended at 4 °C in the assay buffer for carrying out further experiments.

### 3.4. β-Galactosidase Assay

The hydrolysis of β-galactosidase was analyzed by continuously shaking an assay solution containing 1.79 mL of potassium phosphate assay buffer (100 mM, pH 7.0), 0.2 mL of 2.0 mM ONPG, and 100 µL of β-galactosidase (2 U) for 15 min at 40 °C. The product was obtained by terminating the reaction with 1 M Na_2_CO_3_ (2 mL) and measuring it spectrophotometrically at 405 nm. One unit of β-galactosidase activity was defined as the amount of enzyme that liberates 1.0 µmole of *o*-nitrophenol (ε_m_ = 4500 L/mol/cm) per min under standard assay conditions [41].

### 3.5. Physical and Chemical Stability of PVA-AuNPs-β-Galactosidase

The activity of the free enzyme and the enzyme immobilized on PVA-modified AuNPs was analyzed in 100 µL of enzyme (equivalent to 2 U) in various buffers ranging from pH 5.0 to 9.0 which included sodium acetate buffer (pH 5.0), potassium phosphate (6.0, 7.0), and Tris-HCl (pH 8.0, 9.0). The molarity of the buffers used was 100 mM. Since the activity obtained at pH 7.0 was 100%, it was considered as the control for estimating the enzyme activity for the other pH range buffers. Similarly, the stability of the free enzyme and PVA-AuNPs-bound β-galactosidase (100 µL) was demonstrated from 20 to 70 °C in 100 mM potassium phosphate buffer (pH 7). Since the activity obtained at 40 °C was 100%, it was considered as control for estimating the enzyme activity at other temperatures. The effect of galactose (1.0%–5.0%, *w*/*v*) on the activity of the soluble β-galactosidase and the enzyme bound to PVA-modified AuNPs (100 µL) was determined in potassium phosphate buffer (pH 7, 100 mM) at 40 °C for 1 h. The enzyme activity analyzed without the addition of galactose was taken as the control (100%) for calculating the remaining percent activity of the soluble and immobilized enzymes at other concentrations.

### 3.6. Operational Stability of PVA-AuNPs-β-Galactosidase

The operational stability of immobilized enzyme (100 µL) was analyzed under constant stirring by estimating the lactase activity through six consecutive runs according to the procedure described before. The reaction was stopped by sodium carbonate after every run. Immobilized enzyme was obtained by centrifuging at 2000× *g* for 10 min and washed thrice with 100 mM potassium phosphate buffer (pH 7.0). The procedure was repeated with a fresh ONPG solution for analyzing the enzyme activity for each consecutive run. The operational stability of each run/cycle was estimated in terms of relative activity (%) as: Enzyme activity at the end of each cycle/enzyme activity for the first cycle × 100.

### 3.7. Statistical Analysis

The values are expressed as the mean for three independent experiments performed in triplicates with an average standard deviation <5%. One-way ANOVA was used to analyze the data.

## 4. Conclusions

The main objective of immobilizing β-galactosidase for enhancing its stability in biocatalytic processes was achieved herein. For this purpose, AuNPs were synthesized and characterized by XRD, TEM, and UV spectra. The surface of the synthesized AuNPs was modified by polyvinyl alcohol in order to obtain an improved immobilization yield. It also possessed low biodegradability, greater mechanical stability, prolong durability and no side effects for enzyme reactions. The developed modified nanosupport allowed the immobilized β-galactosidase to be reused for several runs and enabled greater resistance against galactose-mediated product inhibition. Additionally, immobilization improved the performance of β-galactosidase against pH and temperature tolerance.

## Data Availability

Not available.

## Sample Availability

Not available.
